# VEGF and Delta-Notch: interacting signalling pathways in tumour angiogenesis

**DOI:** 10.1038/sj.bjc.6604484

**Published:** 2008-09-30

**Authors:** G Thurston, J Kitajewski

**Affiliations:** 1 Regeneron Pharmaceuticals, 777 Old Saw Mill River Road, Tarrytown, New York, NY 10591, USA; 2 Herbert Irving Comprehensive Cancer Center, Columbia University, 1130 St Nicholas Avenue, New York, NY 10032, USA

**Keywords:** Dll4, endothelial, Notch1, Kdr, anti-angiogenesis

## Abstract

Tumour angiogenesis has become an important target for antitumour therapy, with most current therapies aimed at blocking the VEGF pathway. However, not all tumours are responsive to VEGF blockers, and some tumours that are responsive initially may become resistant during the course of treatment, thus there is a need to explore other angiogenesis signalling pathways. Recently, the Delta-Notch pathway, and particularly the ligand Delta-like 4 (Dll4), was identified as a new target in tumour angiogenesis. An important feature in angiogenesis is the manifold ways in which the VEGF and Delta-Notch pathways interact. The emerging picture is that the VEGF pathway acts as a potent upstream activating stimulus for angiogenesis, whereas Delta-Notch helps to guide cell fate decisions that appropriately shape the activation. Here we review the two signalling pathways and what is currently known about the ways in which they interact during tumour angiogenesis.

Solid tumours require the growth of new blood vessels (angiogenesis) to grow (Folkman, 2002). Tumour angiogenesis utilises at least some of the angiogenic signalling pathways that are required during vascular development. Over the past 10 years, these pathways have been recognised as important targets for antitumour therapy, and a number of different approaches to block angiogenic signalling pathways, and thus block tumour growth, have been developed.

At the present time, blocking the VEGF pathway is the best-validated approach to blocking tumour angiogenesis. Studies from a number of years ago showed that the VEGF pathway is central and absolutely essential for early stages of developmental angiogenesis; these findings helped drive the development of VEGF inhibitors for clinical use (Ferrara, 1999). However, unlike developmental angiogenesis, not all tumours are responsive to VEGF blockers, and some tumours that are responsive initially may become resistant during the course of treatment. Thus, there is a need to explore other angiogenesis signalling pathways as therapeutic targets, including those that interact with the VEGF pathway.

One such pathway is Delta/Jagged-Notch signalling system, which is generally involved in cell fate decisions. One particular ligand, Dll4, was recently identified as a potentially important new target in tumour angiogenesis. Other members of this pathway, including Notch receptors, are also required for developmental angiogenesis. The emerging picture is that the VEGF pathway acts as a potent upstream activating stimulus for angiogenesis, whereas Delta-Notch helps to shape that activation appropriately. An important feature of angiogenesis is the manifold ways in which the VEGF and Delta-Notch pathways interact.

In this review, we briefly summarise the components and features of the VEGF and Delta-Notch pathways, including their roles in developmental and tumour angiogenesis. Then we summarise in more detail what is currently known about the ways in which these pathways interact in the vasculature. A deeper understanding of these interactions should lead to better use of agents that block the Dll4-Notch pathway, alone and in combination with currently used inhibitors of the VEGF pathway.

## The VEGF pathway

### Molecular components, general features and functions

The VEGF system has been conserved from zebrafish to man as a signalling pathway that is essential and rather specific to the vascular and haematopoietic systems. In mammals, the system comprises five secreted ligands (VEGF-A, VEGF-B, VEGF-C, VEGF-D and PlGF) and three primary receptors (VEGF-R1, VEGF-R2, VEGF-R3). In general, the ligands are expressed rather broadly and display multiple splice isoforms of varying size and degree of interaction with the extracellular matrix. An important point is that the expression of VEGF-A is induced by hypoxia, which is a common feature of rapidly growing solid tumours, and constitutes a key signal from a tissue calling for more blood vessels and/or increased vascular function. In contrast to the ligands, the receptors show much more restricted cell-type expression. VEGF-R1 and VEGF-R2 are prominently expressed by vascular endothelial cells in addition to expression by selected other cell types. VEGF-R3 is prominently expressed in lymphatic endothelial cells, although it can also be expressed on activated blood endothelial cells (Tammela *et al*, 2005). The ligands show discrete binding to specific receptor(s); for example, VEGF-A interacts with VEGF-R1 and VEGF-R2, whereas PlGF interacts strictly with VEGF-R1. Additional components of the system include the cell-surface proteins, neuropilin-1 and -2, which can serve as coreceptors on endothelial cells for larger isoforms of VEGF-A and VEGF-C. The VEGF receptors are tyrosine kinases that autophosphorylate upon dimerisation by ligand, which in turn activates various downstream signalling pathways. Signals from VEGF receptors drive diverse cellular effects in endothelial cells, including migration, proliferation, cell survival and the expression of downstream genes.

### Genetic deletion in mice and knockdown in zebrafish

Genetic targeting studies in mice have emphasised the essential nature of the VEGF pathway for the development of the blood and lymphatic vascular systems. Genetic deletion of *VEGF-A* or its primary signalling receptor *VEGF-R2* results in early embryonic lethality (approximately embryonic day E9), associated with a near-complete block of haematopoietic and vascular development. Somewhat analogously, deletion of *VEGF-C* results in a severe block in the development of the lymphatic system. Deletion of the main receptor for VEGF-C, *VEGF-R3*, results in embryonic lethality associated with blood vascular defects; however, this receptor becomes essential for the development of the lymphatic system slightly later in development (Tammela *et al*, 2005). Deletion of *VEGF-R1* also results in embryonic lethality, although in this case, the defect is an overabundance of blood vessels. Deletion of other VEGF ligands (*PlGF*, *VEGF-B*, *VEGF-D*) is not lethal and is associated with much more subtle phenotypes. Studies in zebrafish also reveal essential upstream roles for the VEGF system: high-dose morpholino knockdown of VEGF-A in early zebrafish embryos (day 2) results in a near-total absence of vasculature (similar to results in mice), whereas lower dose VEGF-A knockdown results in severe disruption of the dorsal aorta and intersegmental arteries (Nasevicius *et al*, 2000; Lawson *et al*, 2002).

### Blockade of the VEGF pathway in tumours

The VEGF pathway was identified as a target for antitumour therapy more than 15 years ago, and early studies showed that blocking the VEGF-A ligand with a monoclonal antibody could result in strong suppression of tumour growth (Ferrara, 1999). Since these initial studies, many different approaches have been taken to block the VEGF pathways, focusing on VEGF-A ligand and VEGF-R2 receptor (Duda *et al*, 2007). Various preclinical studies have shown that successful blockade of the pathway in tumour-bearing mice results in vessel pruning and reduction of angiogenic sprouting, which produce a ‘normalisation’ of the aberrant tumour vessels (Duda *et al*, 2007). The reduced tumour angiogenesis leads to reduced tumour growth in a broad variety of tumour types. These preclinical results have been validated by successful phase 3 clinical trials of anti-VEGF agents in several types of cancers, including colorectal, renal cell carcinoma and breast cancer. Studies in numerous cancer indications are currently ongoing.

## The Delta/Jagged-Notch pathway

### Molecular components, general features and functions

Notch is a fundamental signalling pathway in many mammalian cell types undergoing differentiation, primarily acting to regulate cell fate determination. Delta-Notch signalling is a form of cell–cell communication, helping a group of similar cells to integrate contextual information and steer down separate pathways of differentiation. One mechanism, referred to as lateral inhibition, involves presentation of a Notch ligand (Delta or Jagged) to an adjacent Notch receptor cell, resulting in activation of the Notch pathway in one cell and suppression in the adjacent cells. The end result locks the cells into distinct cellular fates; one promoted by Notch signal activation and the other dependent on lack of Notch signalling. Notch receptors are distinct in that they operate both on the cell surface to bind ligand and within the nucleus as transcriptional modulators (Mumm and Kopan, 2000). The pathway in mammals is comprised of a conserved family of four transmembrane receptors (Notch1–4). In the vasculature, Notch1 and Notch4 function in developing endothelium, whereas Notch3 is critical for smooth muscle cell differentiation. Mammals utilise five membrane-bound Notch ligands (Delta-like 1, 3, 4, Jagged1 and -2). Those ligands implicated in the process of angiogenesis include Delta-like 1 (Dll1), Delta-like 4 (Dll4) and Jagged1. Receptor–ligand interactions between adjacent cells first trigger the metalloproteinase ADAM (a disintegrin and metalloproteinase) to cleave the Notch extracellular region and then trigger *γ*-secretase-mediated generation of the Notch intracellular domain (NICD). Subsequently, NICD translocates to the nucleus, where it interacts with the CSL (RBPJ) transcription factor to induce multiple downstream targets, including a family of basic helix-loop-helix proteins, the Hes and Hey genes (Mumm and Kopan, 2000).

### Genetic deletion in mice and knockdown in zebrafish

Mice with targeted deletions of Notch family genes exhibit diverse vascular defects, mostly affecting the process of vascular remodelling and arterial specification. *Notch1*, *Dll4* and *CSL* mutations result in the most severe vascular disruptions. The Notch1 null mice die due to profound defects of angiogenic vascular remodelling, shown most obviously as a failure of proper formation of the major arteries. The mice form a primary vascular plexus, indicating that Notch signals are not required for vasculogenesis and early angiogenesis; however, this plexus fails to remodel into an organised network of larger and smaller vessels (Krebs *et al*, 2000). Mice lacking the *Notch4* gene develop normally; however, Notch1/Notch4 double mutants show more severe vascular defects than mice lacking only *Notch1*, suggesting that Notch1 and Notch4 have partially redundant functions (Krebs *et al*, 2000). Proper vascular development requires appropriate Notch activity, as mice with an activated allele of *Notch4* (Notch4/int3) die *in utero* and display severe vascular anomalies, reminiscent of those seen in *Notch1* null mice (Uyttendaele *et al*, 2001). Similarly, targeted endothelial expression of INT3 produces reversible arteriovenous defects in adult mice (Carlson *et al*, 2005). Jagged1 deletion also results in lethality due to haemorrhage and failure in angiogenic remodelling (Xue *et al*, 1999). In support of its role as the primary endothelial cell Notch ligand, heterozygous deletion of *Dll4* is lethal. Vascular defects in *Dll4* mutants include arteriovenous malformations, a lack of vascular remodelling and collapse or incomplete formation of the major arteries (Duarte *et al*, 2004; Gale *et al*, 2004; Krebs *et al*, 2004). In rare-surviving *Dll4* heterozygous mice, defects were also noted in sprouting angiogenesis in the neonatal retinal vessels (Lobov *et al*, 2007; Suchting *et al*, 2007); these defects are understood in terms of a failure for local cell fate decisions in the leading front of angiogenic vessels (see below). In zebrafish, Delta-Notch signalling has been shown to be important for the differentiation of arterial endothelial cells and proper formation of the major arteries (Lawson *et al*, 2001). In addition, Notch signalling is also required for proper control of angiogenic sprouting of, for example, the intersomitic vessels (Leslie *et al*, 2007; Siekmann and Lawson, 2007).

### Blockade of Dll4 in tumours

As could be expected from its role in developmental angiogenesis, the Delta-Notch pathway is also important in tumour angiogenesis. Several studies have documented the expression of Notch components in tumour vessels, most notably Dll4. Studies of tumours in mice and humans have shown that Dll4 is strongly expressed in tumour blood vessels compared to adjacent normal vessels. For example, in human tumours, Dll4 expression was localised to the blood vessels of clear-cell renal tumours (Mailhos *et al*, 2001) and was at nine-fold higher levels than in normal kidney tissue (Patel *et al*, 2005). In keeping with the studies described above, expression of Dll4 in tumour vessels appears to be directly regulated by VEGF. For example, blockade of VEGF in tumour-bearing mice results in a rapid and profound reduction of Dll4 expression by the tumour blood vessels (Noguera-Troise *et al*, 2006). Conversely, the levels of Dll4 expression in tumours correlate with the levels of VEGF expression (Patel *et al*, 2006). Thus, the high levels of Dll4 expression on tumour vessels may be a result of relatively high levels of VEGF signalling in these vessels compared to most normal vessels.

The striking pattern of Dll4 expression in tumour vessels prompted several groups to target Dll4-Notch activity and provided insight into a role for Dll4-Notch in regulating VEGF-induced vascular sprouting. Local or systemic treatment of mice with Dll4-Notch inhibitors caused overgrowth of a non-functional tumour vasculature. However, strikingly, blockade of Dll4-Notch resulted in a growth inhibition in a variety of established human and rodent tumour models (Noguera-Troise *et al*, 2006; Ridgway *et al*, 2006; Scehnet *et al*, 2007). The reduced growth was associated with an increase in tumour vessel density and increased vessel sprouts with numerous interconnecting branches. Even though the tumours were smaller due to Dll4 blockade, tumours were more hypoxic, apparently containing non-functional vessels (‘abnormalisation’ – Thurston *et al*, 2007).

Other Notch ligands may also influence tumour angiogenesis; for example, one study suggests an angiogenic role for Jagged1 in head and neck tumours (Zeng *et al*, 2005). A similar conclusion was reached in a study of a novel Notch inhibitor, the Notch1 decoy, which has the ability to block Notch signalling via several different Notch ligands, including both Dll4 and Jagged1. The Notch1 decoy was shown to block angiogenesis in both a VEGF-driven dermal angiogenesis model and a xenograft model of mammary tumour growth (Funahashi *et al*, 2008). It remains to be established which ligands are most critical for the antiangiogenic effect of the Notch1 decoy.

## Interactions of VEGF and Delta-Notch pathways

### Genetic evidence for interaction between VEGF and Delta-Notch pathways

An initial appreciation of the relationship between the VEGF pathway and the Delta-Notch pathway came from studies of vascular development in zebrafish. Several studies have shown that loss of Notch signalling, for example with morpholino knockdown of Notch receptors or in mutants of the downstream gene gridlock, leads to defective development of the arterial vessels (Zhong *et al*, 2001). Subsequently, VEGF was shown to act upstream of Notch in determining arterial cell fate in vascular development (Lawson *et al*, 2002). By analogy to the studies of VEGF activity in cultured endothelial cells, it is possible that VEGF signalling leads to increased Dll4 and Notch expression, in turn leading to productive Notch signalling and thus arterial specification characterised by expression of a defined set of arterial genes. However, it is not clear whether a VEGF signal in isolation can induce *de novo* expression of Notch components (Lawson *et al*, 2002). More work will be needed to fully define the biochemical link between the VEGF pathway and the Delta-Notch pathway in this setting (Siekmann *et al*, 2008).

### VEGF increases Dll4 expression

Studies in several systems, discussed here, establish that VEGF regulates the expression of Notch signalling components. Studies with cultured endothelial cells reveal several ways in which the VEGF and Dll4-Notch pathways interact. Importantly, VEGF increases Dll4 expression. Treatment of cultured endothelial cells with VEGF results in increased expression of Dll4 mRNA and protein (Patel *et al*, 2005; Hainaud *et al*, 2006; Ridgway *et al*, 2006). Vascular endothelial growth factor is a classic hypoxia response gene; thus the induction of Dll4 can occur indirectly in situations of hypoxia, via induction of VEGF. In addition, Dll4 expression by endothelial cells may be directly upregulated by hypoxia, possibly via Hif1-*α* and hypoxia response elements in the Dll4 promoter (Patel *et al*, 2005; Diez *et al*, 2007).

The expression of Dll4 is also increased by VEGF *in vivo*. Dll4 is strongly expressed by endothelial cells of sprouting angiogenic vessels, which are commonly responding to VEGF signals. For example, in the developing murine retina, a VEGF gradient emanating from the avascular regions of the retina is essential for the sprouting angiogenesis. These growing vessels, and in particular the ‘tip cells’, are associated with strong expression of Dll4 (Hellstrom *et al*, 2007; Lobov *et al*, 2007). Blocking VEGF, by intravitreal injection of soluble VEGF receptors, results in decreased sprouting and decreased expression of Dll4 on the retinal vessels (Suchting *et al*, 2007). Conversely, intravitreal injection of VEGF protein increased expression of Dll4 in the retina (Lobov *et al*, 2007). Furthermore, in an oxygen-induced model of retinal angiogenesis (OIR), Dll4 expression is also increased on the newly forming retinal vessels (Lobov *et al*, 2007). Again, specific blockade of VEGF blocks the increased expression of Dll4 on the newly forming retinal vessels in the OIR model. It is noteworthy that blocking VEGF in this model does not reduce expression of Dll4 on arteries, indicating that other signals can also induce expression of Dll4.

**Figure 1:**
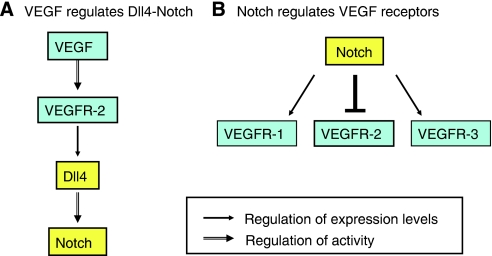
Schematic diagram summarising the different ways in which the VEGF and Notch pathways interact. (**A**) VEGF stimulus, acting via VEGF-R2, increases expression of Dll4 on endothelial cells, which in turn activates Notch receptors on adjacent endothelial cells. (**B**) Activated Notch receptors on endothelial cells can in turn positively (VEGF-R1, VEGF-R3) or negatively (VEGF-R2) regulate the expression of VEGF receptors in those cells.

Similar associations between VEGF signalling, growing vessels and Dll4 expression have been found in tumour vessels (Mailhos *et al*, 2001; Patel *et al*, 2005; Noguera-Troise *et al*, 2006). As described above, VEGF drives a key pathway for tumour angiogenesis in many models, and ongoing VEGF signalling is often required for sprouting angiogenesis in tumours. Increased expression of Dll4 in tumour vessels has been reported in several mouse tumour models (Mailhos *et al*, 2001; Noguera-Troise *et al*, 2006) and in human tumours (Patel *et al*, 2005). For example, Dll4 expression was found to be particularly strong on the growing front of vessels as tumours become vascularised (Noguera-Troise *et al*, 2006). Conversely, blockade of VEGF in tumours resulted in a rapid decrease of Dll4 expression in tumour vessels (Noguera-Troise *et al*, 2006), showing that a significant portion of the Dll4 expression in the growing tumour vessels requires ongoing VEGF signalling. Thus, in tumours, VEGF can act to regulate the expression of Dll4, as schematised in Figure 1A. Other ligands for Notch, particularly Jagged1, have also recently been associated with angiogenic endothelial cells (Sainson *et al*, 2008). It will be very important to determine whether the different ligands provide the same Notch signalling activity in angiogenic endothelial cells.

### Dll4-Notch signalling affects expression of VEGF receptors

Separate studies have suggested that Notch signalling can alter expression levels of all three VEGF receptors, schematised in Figure 1B. The best-characterised case is that of VEGF-R2, which is downregulated by either Notch or Hey1 (a Notch target gene). Downregulation of VEGF-R2 has been observed following activation of Notch in cultured endothelial cells (Taylor *et al*, 2002). Reciprocally, increased VEGF-R2 levels were observed in vessels of Dll4 heterozygous mice or as a result of Dll4 blockade (Suchting *et al*, 2007). This important finding suggests that Notch can provide negative feedback to reduce the activity of the VEGF/VEGF-R2 axis. In cultured endothelial cells, downregulation of VEGF-R2 may explain the reduced endothelial proliferation and migration seen as a result of Notch signal activation (Taylor *et al*, 2002). *In vivo*, downregulation of VEGF-R2 has been proposed as a mechanism to permit local differentiation of cells within a zone of VEGF-driven angiogenesis: high levels of VEGF induce Dll4 in an endothelial ‘tip cell’, which in turn provides a Notch signal and decreased VEGF-R2 in downstream ‘stalk cells’ (Roca and Adams, 2007). Through this pathway of local cell differentiation, the tip cells respond robustly to VEGF whereas the stalk cells respond in a more muted manner.

Two recent reports suggest that VEGF-R1 expression may be increased by Notch signalling. Reduced Notch activity, such as in Dll4 heterozygous mice, resulted in reduced VEGF-R1 expression, in addition to increased VEGF-R2 expression (Suchting *et al*, 2007). In addition, Dll4-Notch activity resulted in increased expression of both the signalling form of VEFR-1 and the soluble form of VEGF-R1 (sFlt1) in cultured endothelial cells (Harrington *et al*, 2008). VEGF-R1 is a less potent signalling receptor than VEGF-R2; thus it is not clear whether changing it's coexpression with VEGF-R2 would tend to increase, decrease or change the character of the signal.

A recent study describes the VEGF-R3 gene as a direct transcriptional target of the Notch signalling pathway, with NICD and the CSL transcription factors binding directly to sites within the human and murine VEGF-R3 promoters (Shawber *et al*, 2007). In addition to showing a dramatic increase in VEGF-R3 expression, Shawber *et al* (2007) also showed an alteration of endothelial VEGF responses as a result of Notch signal activation. Notch signalling increased the cellular responses to VEGF-C, presumably via upregulation of VEGF-R3, and decreased the response to VEGF-A, via downregulation of VEGF-R2. An analogous regulation of VEGF-R3 was also apparent in mouse embryos following increased or decreased Notch activity. Finally, although heterozygous Notch1 or VEGF-R1 mice are viable, doubly heterozygous Notch1−/+VEGFR1−/+ embryos often die due to vascular problems. The regulation of VEGF-R3 by Notch is likely complex, as not all vascular beds showed increased VEGF-R3 levels in response to Notch signalling. In zebrafish, Notch has been proposed to downregulate the VEGF-R3 homologue, zFlt4, during arterial specification and in the angiogenic tip cells of intersomitic vessels (Siekmann and Lawson, 2007). Thus, Notch may interact with the VEGF-R3 promoter in a context- and cell type-specific fashion leading to different outcomes in promoter regulation. In sum, these results suggest a complex interplay between Notch activity and expression of VEGF receptors, resulting in changes in how a particular endothelial cell will respond to VEGF signals.

### Combination blockade of VEGF and Dll4 is more effective in some tumours

An exciting opportunity has arisen because recent studies have shown that blockade of Dll4 can have potent antitumour effects on tumours that are resistant to VEGF inhibition (Noguera-Troise *et al*, 2006; Ridgway *et al*, 2006). For example, the growth of a sarcoma model that is very resistant to VEGF blockade was strongly inhibited by blockade of Dll4-Notch (Noguera-Troise *et al*, 2006). In addition, the tumour vessels in this model showed dramatic disorganisation and dysfunction following Dll4-Notch blockade. Furthermore, simultaneously blocking both VEGF and Dll4 can have more potent effects in a variety of tumour models than blockade of either factor alone (Ridgway *et al*, 2006; I Noguera-Troise *et al*, unpublished results), including this sarcoma model. Previous studies have shown that individual blockade of VEGF and Dll4-Notch produces very different effects on the morphology and function of tumour vessels (above, also Thurston *et al*, 2007); however, to date, there is little information on the effects of blocking both pathways in combination.

Although these observations raise the possibility of using combination antiangiogenic therapies against the two pathways, much remains to be learned regarding how to best exploit this approach clinically. The factors produced by the tumour or expressed within the tumour vessels that confer sensitivity to Dll4-Notch blockade are unknown. To be fair, it must be said that the corresponding factors that confer sensitivity to VEGF blockade are also largely unknown, despite intense investigation for a decade. A useful starting point may be to assume that high levels of Dll4-Notch are in some way correlated with sensitivity to blockade. Thus, further information from clinical specimens on the levels of the various components of the VEGF and Delta-Notch pathways in different tumour types will be very useful.

Mechanistically, the observation of potent combination effects is difficult to fit into a simple model in which VEGF signalling induces Dll4-Notch activity in the vasculature, which subsequently helps to downregulate VEGF pathway activity in a neat feedback loop. It is clear that although this model may be fundamentally correct, other complexities are in play. For example, VEGF signalling induces a number of different downstream pathways and thus Dll4-Notch represents only one output. Conversely, Dll4 is also apparently upregulated by pathways other than VEGF, including in response to Notch signalling itself (Shawber *et al*, 2003; Ridgway *et al*, 2006). An interesting approach might be to consider whether Dll4 blockade is sensitising tumour vessels to VEGF blockade or vice versa. Alternatively, the combined blockade could be acting on separate subsets of tumour vessels within a heterogeneous network. Future studies to document the effect of combination blockade on the morphology and function of tumour vessels, combined with studies of the interaction of the pathways in cultured endothelial cells, are needed to shed further light on this issue.

## Discussion and future directions

The formation of a hierarchical network of vessels requires the coordinated interplay of various signalling pathways. Even tumour vessels, which are structurally abnormal and poorly functional, can be rendered less efficient by interfering with vascular signalling pathways. As described in this review, two important signalling pathways with distinct roles in vascular development, namely the VEGF and Delta-Notch pathways, interact at several levels to help generate a hierarchical vessel network. Some of the interaction appears to be at the level of gene transcription; for example, the activation of VEGF-R2 can lead to increased expression of Dll4. However, given the complexity of both the pathways, other levels of cross-control are likely and need to be explored.

**Figure 2:**
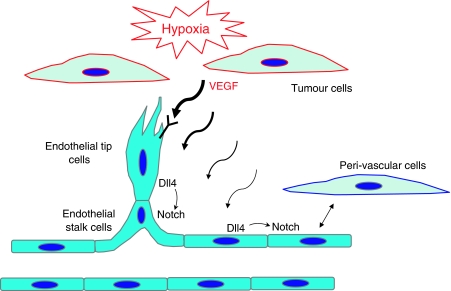
Comparison of the roles of VEGF and Delta-Notch pathways in angiogenesis and vascular development. Vascular endothelial growth factor from hypoxic tumour cells and surrounding tissue provides a signal to endothelial cells (via cell-restricted VEGF receptors) that calls for an increase in vascular function. The Delta-Notch pathway (particularly Dll4) acts within the vasculature to help the endothelial cells respond appropriately to the activating VEGF signal. At least two settings in the developing vasculature apparently utilise VEGF as a driving signal and Delta-Notch to make cell fate decisions: the specialisation of tip cells and stalk cells within the growing front of angiogenic vessels and the specification of arterial cell fate.

Conceptually, the VEGF system provides a driving signal from surrounding tissues to endothelial cells (via cell-restricted VEGF receptors) that calls for an increase in vascular function. However, not all cells should respond equally to VEGF, and thus cell fate decisions are essential for generating an efficient response. Subsequently, the Delta-Notch pathway (particularly Dll4 and Notch1) acts among the endothelial cells to respond appropriately to the activating VEGF signal (Figure 2). In turn, an appropriate vascular response will provide increased vascular function and ultimately reduce the VEGF driving signal. Studies to date have indicated at least two settings in the developing vasculature in which VEGF drives a process and Delta-Notch helps to make cell fate decisions within that process; in particular, the specification of arterial cell fate and the specialisation of tip cells and stalk cells within the growing front of angiogenic vessels. It will be important to determine whether two different cell fate settings are applicable to the very chaotic process of tumour angiogenesis.

Further studies are needed to examine the interaction of the pathways in various tumour settings. We are just beginning to explore how we can take combinatorial advantage of the VEGF and Delta-Notch pathways for treating tumours, and a better conceptual framework within the context of tumour angiogenesis would help guide the experiments. Another issue will be to clarify some of the disparate results on the regulation of VEGF receptors by Delta-Notch activity: does VEGF receptor expression depend on endothelial cell phenotype, on the nature of Notch signal or on the presence of additional stimuli? Another important topic will be to determine how some of the key downstream Notch target genes (for example, ephrinB2) affect tumour vessels in the presence of high VEGF levels. Another potentially fruitful approach will be to examine whether other types of endothelial cell fate decisions in development are guided by Delta-Notch. We are at an exciting time for the convergence of basic vascular biology and tumour therapy.
